# Electronic Melting of Silicon in Nanostructures using X-ray Forbidden Bragg Reflections

**DOI:** 10.1007/s11664-025-11781-2

**Published:** 2025-02-17

**Authors:** Ian Robinson, David Yang, Longlong Wu, Hyunjung Kim, Sung Soo Ha, Sungwook Choi, Changyong Song, Junha Hwang, Seung-Phil Heo, Jaeku Park, Intae Eom, Sunam Kim

**Affiliations:** 1 Brookhaven National Laboratory, Upton, NY 11973 USA; 2 London Centre for Nanotechnology, University College, London, WC1E 6BT UK; 3 Center for Ultrafast Phase Transformation, Sogang University, Seoul, Korea; 4 Dept. of Physics, POSTECH, Pohang, Korea; 5 PAL-XFEL, Pohang Accelerator Laboratory, Pohang, Korea

**Keywords:** Laser excitation, silicon, valence electrons, forbidden reflections, non-thermal melting

## Abstract

We carried out a short beamtime at the Pohang Accelerator Laboratory x-ray Free Electron Laser to perform a pump-probe (PP) laser excitation diffraction experiment on the silicon (222) forbidden Bragg peak. To limit the x-ray penetration, we used a “device layer” silicon film wafer bonded to a silicon substrate. The sample, specially fabricated by MEMC Electronic Materials, had a Si(100) substrate bonded to a 170 nm Si(100) film rotated at 45° for crystallographic isolation. A second sample was reactive-ion-etched down to 52 nm thickness. In the silicon lattice, the covalent bonds are seen exclusively at the 222 reflection. Upon laser excitation, these electrons are expected to be excited to the valence band on femtosecond electronic time scales. The Si(222) reflection is therefore expected to be extinguished on this fast time scale, while the electron–phonon coupled acoustic response is determined by the lattice dynamics. The latter is determined by the speed of sound over the device thickness, which is in the mid-picosecond range.

## Introduction

The ultrafast time domain reveals a new dimension to materials physics: a rich variety of metastable materials can become accessible in the transient period following laser excitation from the ground state. Time can be added as a new dimension to the general classification of a material by its phase diagram, which displays its properties as a function of external state variables, typically temperature and pressure. Examples include a “hidden phase” of Nd_0.5_Sr_0.5_MnO_3_ [1], a transient phase of elemental gallium lasting tens of picoseconds [2], transient superconductivity all the way up to room temperature [3] and an electronically ordered excited phase of C_60_ [4].

The advent of x-ray free electron laser (XFEL) facilities, such as the Pohang Accelerator Laboratory (PAL)-XFEL, opens the possibility of performing pump-probe (PP) x-ray diffraction experiments. The synchronization of a femtosecond laser pulse exciting the sample with a similar duration x-ray pulse measuring its diffraction is adjusted to map out the time delay. This allows the fine structural details accessible by x-ray diffraction to be examined on a time scale of femtoseconds. In any crystalline material, there are two expected time scales which can be probed by PP methods, corresponding to electron and lattice dynamics. The laser pump pulse (typically 10 fs long) directly excites electrons which migrate rapidly in metals and semiconductors, typically at the Fermi velocity around 10^6^ m/s, traversing a 100 nm-sized sample in 100 fs. However, x-ray diffraction experiments normally do not see these itinerant electrons, but are sensitive to lattice displacements. The electrons are coupled to the crystal lattice based on the widely used two-temperature model (TTM) [5, 6]. The TTM is just the first approximation that the dynamics can be separated into two thermodynamic reservoirs and has been generalized in more complicated systems. The electron–phonon coupling occurs on a later time scale, typically 1 ps, with a cross-section given by the electron–phonon coupling constant. Phonons involve lattice displacements which can be seen by the X-ray probe [7]. They propagate at the speed of sound, typically 10^3^ m/s, traversing a 100 nm-sized sample in 100 ps. The difference in time scale of three orders of magnitude allows for the easy separation of electronic and phonon-mediated processes in solids.

**Fig. 1:**
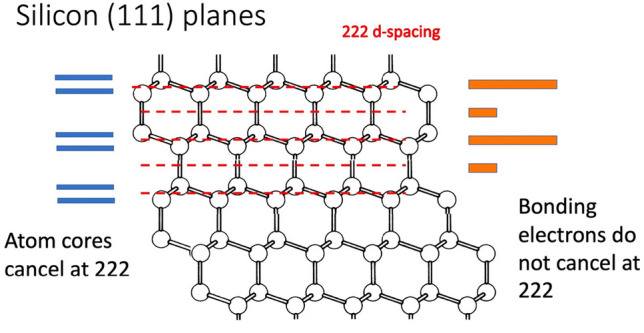
Illustration of how the 222 reflection from the diamond lattice sees a direct contribution from the four sp^3^ valence electrons, but complete cancellation of the structure factor from the spherical core electrons.

The goal of our experiment was to measure the femtosecond temporal response of the bonding electrons in silicon and, by inference, other diamond lattice crystals. Because of the two-atom-per-unit-cell basis in the Fd-3 m space group of diamond and the tetrahedral shape of the sp^3^ bonding electrons, there exists an accessible set of “forbidden” Bragg reflections which can probe these alone, indexed as 222-type, illustrated in Fig. 1. Thermal vibrations also follow this symmetry feature of the space group by appearing in the same structure factors. However, the thermal processes involve the response of the atomic cores to the electronic structure, so are expected to follow on a significantly slower time scale.

**Fig. 2:**
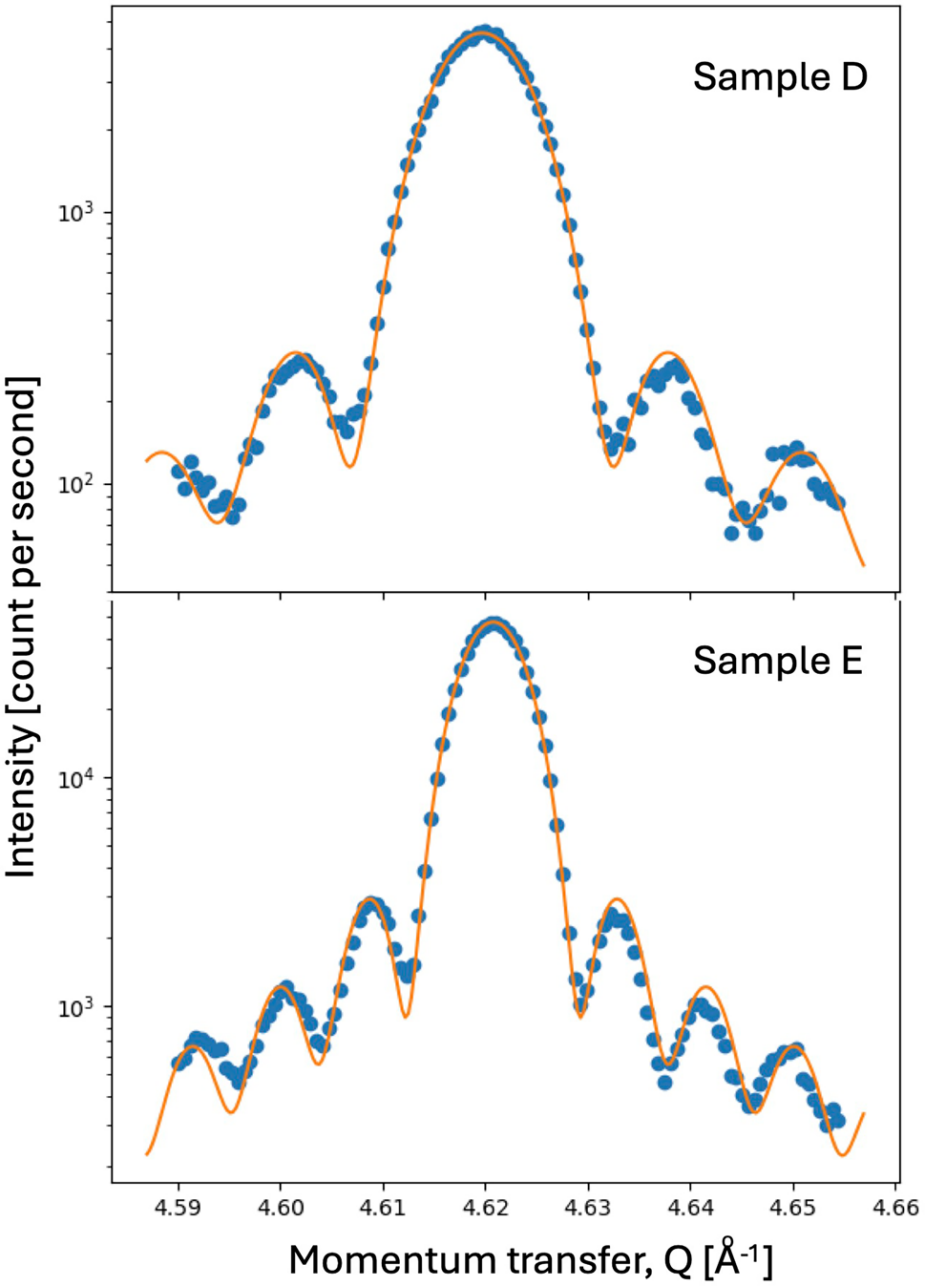
Radial scans of the 004 peak of the RIE-etched SOI films, samples D and E. Log intensity versus momentum transfer, Q, measured on a Bruker diffractometer before the experiment. The fits to a (sinc)^2^ function characteristic of the structure factor of a thin film are superimposed. The fringe spacings determine the film thickness. Sample D (6 s before etching through) is 52 nm thick. Sample E (8 s before etching through) is 75 nm thick from the fringe analysis.

For better agreement between the penetration depth of a few nanometers for the laser pump [8] and the X-ray probe in silicon, we used a fabricated nanostructure specially provided by MEMC Electronic Materials, Inc. (St. Peters, MO, USA). The sample was a Si(100) substrate bonded to a 170 nm Si(100) film rotated 45° for crystallographic isolation. Wafer bonding is used commercially to create silicon-on-insulator (SOI) device layers with higher mobility. Two more samples, D and E, were prepared by thinning the first using a reactive ion etch (RIE) down to 52 nm and 75 nm thickness, respectively. The etch rate was calibrated on sacrificial samples and used to stop the RIE a few seconds before breaking through the film into the substrate. Radial x-ray diffraction scans of the two samples, shown in Fig. 2, show the thin-film fringes expected for well-defined samples, used to determine the thickness. At PAL-XFEL, we used PP measurements in an attempt to spatially determine local variations of these processes associated with the film thickness with the observed phonon population. While the electronic process is highly local, the phonons are sensitive to strains present in the crystal, which become strong for a thin film.

This kind of PP study will help us understand non-thermal melting [9] of the electronic structure in a material, which precedes the disruption of its atomic positions; this is important in covalently bonded crystals with relatively large atomic spacing, such as diamond-lattice semiconductors. The direct ultrafast excitation of the bonding electrons is expected to significantly modify the phonon dispersion relation by hardening the bonds in metals and semiconductors, predicted theoretically [10] and observed in ultrafast electron [11] and recent x-ray [12] diffraction experiments on Au thin films. The electronic redistribution pathway during optical excitation of Si couples to the structure factor of its “forbidden” 222-type reflection. The TTM says that the laser directly excites the electrons within a few tens of femtoseconds [13]. Electron–phonon coupling then transfers this energy to the crystal lattice ions on a picosecond time scale leading to the thermal response of the lattice [10].

Theoretical predictions are very different for different types of material. Density functional theory (DFT) calculations of the phonon spectrum for Si at T_electron_ = 26,000 K (or k_B_T_electron_ = 2.15 eV) show significant softening of the optical branches from 16 THz to 12 THz [10], which would apply during the first picosecond of the TTM model when only the electrons are excited. At a more detailed level, we could use different reflections to distinguish between the electron–phonon coupling and the direct change in the interaction forces seen in the bonds between the atoms. The bonding electrons, which are seen directly by the “forbidden” 222-type reflection, are expected to change on femto- to picosecond time scales while T_electron_ is still high in the TTM.

Si has strong covalent bonding and relatively low density. Optical excitation of Si excites the valence electrons, visible directly in the structure factor of its “forbidden” 222-type reflections, which are sensitive mainly to the bond charges and partially to the asymmetry of the vibrations. There are two Si atoms per primitive unit cell of the crystal lattice with opposite orientation of their bonds, shown in Fig. 1. Along the 111 axis, the symmetric core electrons have a 3:1 layer spacing, causing cancellation of the diffraction intensity at the 222 reflection, while the bonding valence electrons are spread out in between the cores. At the 222 reflection, the scattering from the six valence electrons (per unit cell) within the double layers is not cancelled by the two electrons in the bond in between them, resulting in an allowed contribution to the intensity. The 222 structure factor is (only) 10^3^ times weaker than the 111 because it comes from the bonding sp^3^ electrons, not the Si cores [14], as illustrated in Fig. 1. Using pump-probe X-ray diffraction at the Si(222) reflection, we are able to record this expected electronic depletion effect. Some of the laser-excited electrons will cross the bandgap to fill antibonding orbitals, which will drive atomic displacements that will contribute to the 222 structure factor after a time delay. However, according to DFT calculations, the electron density found in antibonding orbitals in Si is less than 20% of the bonding orbitals [15], so this should be clearly distinguishable.

We started the experiment with optical excitation at 800 nm, which is above the bandgap for Si, so an efficient redistribution of the bonding electrons is expected at short times. This excitation pumps electrons into the conduction band which will rapidly diffuse to the bottom of the band. Based on the TTM, they will eventually start coupling to the lattice, which will lead to heating. Meanwhile, the bonding electrons will be seen directly in the forbidden 222 (and equivalent) Bragg peaks, which are expected to respond rapidly as the electrons become excited and then recover on an intermediate time scale which will be measured through the 222-type Bragg peak intensity.

We performed the experiment at the PAL-XFEL facility to have the necessary time resolution attributed to the 100 fs-duration x-ray pulses. XFEL facilities are relatively new and are not yet as reliable as synchrotron x-ray facilities are today. Because a large staff is required to operate the facility, the beamtime runs are hard to justify and tend to be rather short. This makes complete studies of material samples quite challenging. In our case, we were allotted three 12-h shifts and had problems with the LINAC laser for the first 6 h and could only align without exact PP overlap by the end of the first day.

**Fig. 3:**
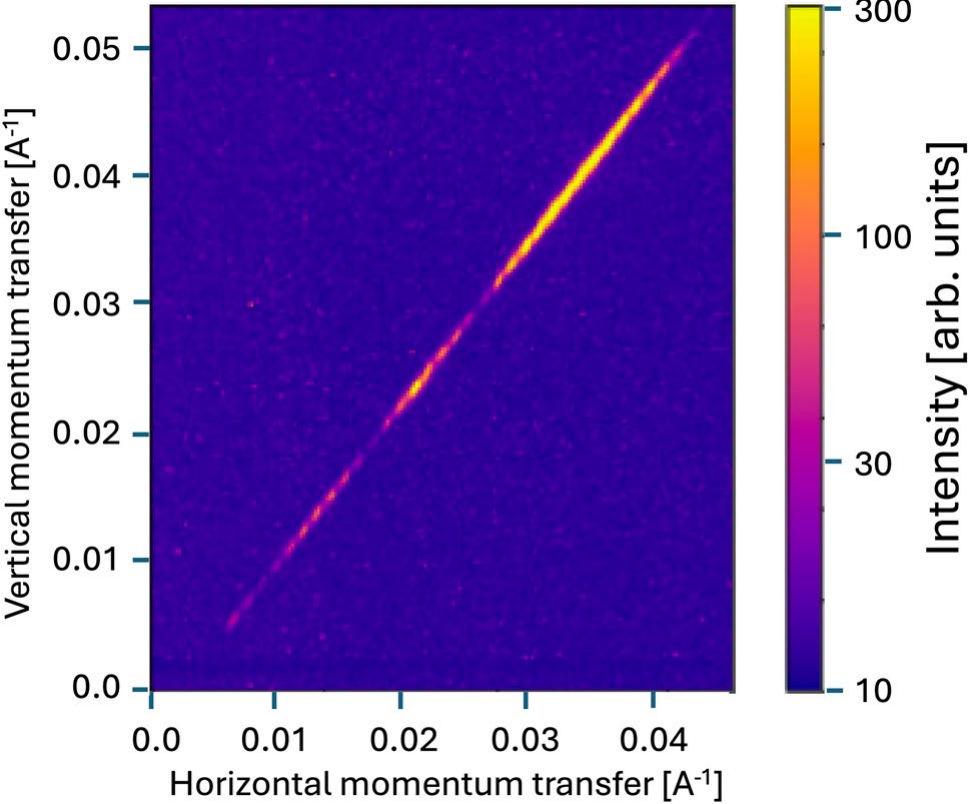
Three hundred shots accumulated from the 222 peak of the 52 nm-thick sample D taken with the sample in a helium atmosphere on the Jungfrau detector located 1.38 m away. The detector images were summed over a rocking scan of width 0.03° with a step size of 0.007°; the fine fringes, just visible as a modulation, are due to the finite step size. The large fringes correspond to the structure factor of the thin film shown in Fig. 2.

The NCI-CXI station of PAL-XFEL has a full six-circle diffractometer with the x-ray and laser beams overlapped at its center of rotation. The incident x-ray beam was focused using compound refractive lenses (CRL), establishing an x-ray spot size of about 20 × 20 µm. The wavelength was 1.278 Å, with a measured bandwidth of 1.1 × 10^−4^ set by the double-crystal monochromator. A Jungfrau detector (Dectris) with a solid mount and a vacuum flight path was placed on the diffractometer arm at a distance of 1.38 m. The laser focus was 100 × 100 μm. The pump laser excitation and probe XFEL beams ran at 30 Hz. The laser to x-ray PP temporal overlap was established using a long time-scan on a Bi crystal. We used the “spec” diffractometer control program to define an orientation matrix for the sample to locate the 222 Bragg peak. The 222 peak for the 170 nm SOI sample was measured with 10^−3^ x-ray attenuation using absorbers, later reduced to 10^−2^ and eventually 8 × 10^−2^ for sample D (52 nm thick). Nice diffraction fringes were seen on sample D, shown in Fig. 3. No x-ray damage was detected even at this attenuation level.

**Fig. 4:**
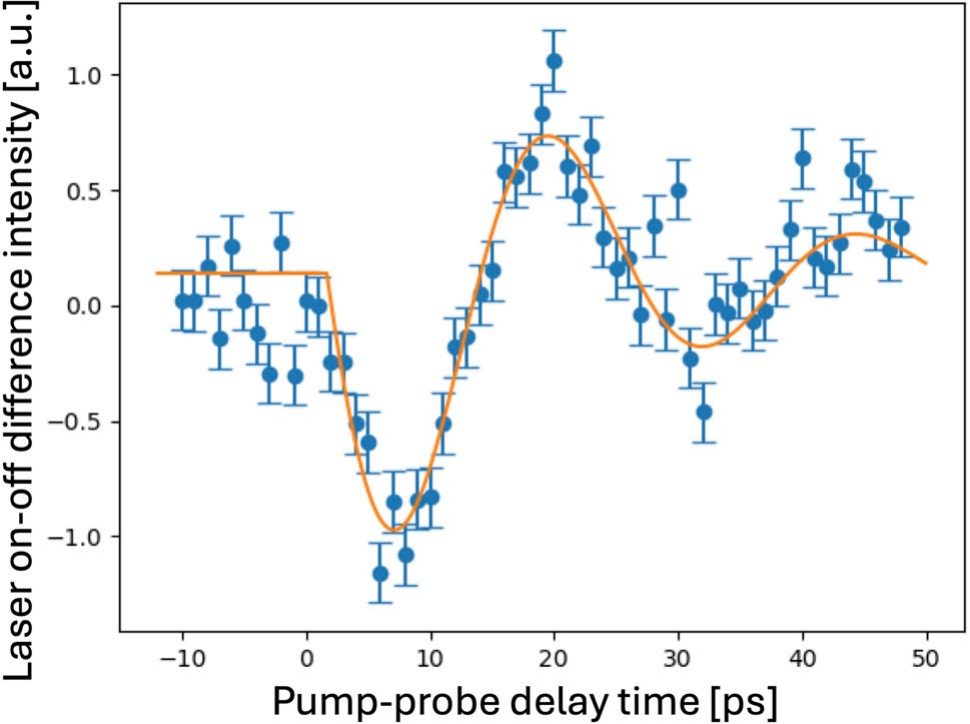
Integrated intensity of the Si(222) reflection of the 170 nm sample versus pump-probe delay. The laser fluence was 50% (250 mJ/cm^2^). The signal shown is the difference between laser-on and laser-off measurements. The fit is to a damped sine wave with a 1.66 ps time offset, a 24.8 ps period, and a 20 ps damping time constant.

PP delay scans with the 800 nm laser excitation did not result in any visible changes to the 222 peak. We switched to 400 nm pulses by inserting a second-harmonic crystal, but we had to repeat the PP overlap using the Bi crystal. The optical absorption length of silicon at 400 nm is about 100 nm and many microns at 800 nm [8]. Hot electrons will be generated over this depth; however, the laser-excited electrons should migrate rapidly to fill the sample, as explained above. After partly removing the neutral-density (ND) filter to produce a strong fluence, we found a nice acoustic response, shown in Fig. 4, with oscillations of 24.8 ps period; 40 ps was expected from the longitudinal speed of sound, 8433 m/s, traversing a thin silicon film with thickness of 170 nm [16]. The acoustic response of a crystal to a laser impulse is expected to produce a “breathing mode” with expansion and compression of the crystal lattice, which has been seen in previous experiments [7]. Figure 4 shows oscillations in the peak intensity. This occurs because we are measuring an off-specular 222 reflection with a very narrow rocking curve. The change in the lattice constant causes a change in angular position on the rocking curve in the horizontal direction which results in a drop of intensity.

**Fig. 5:**
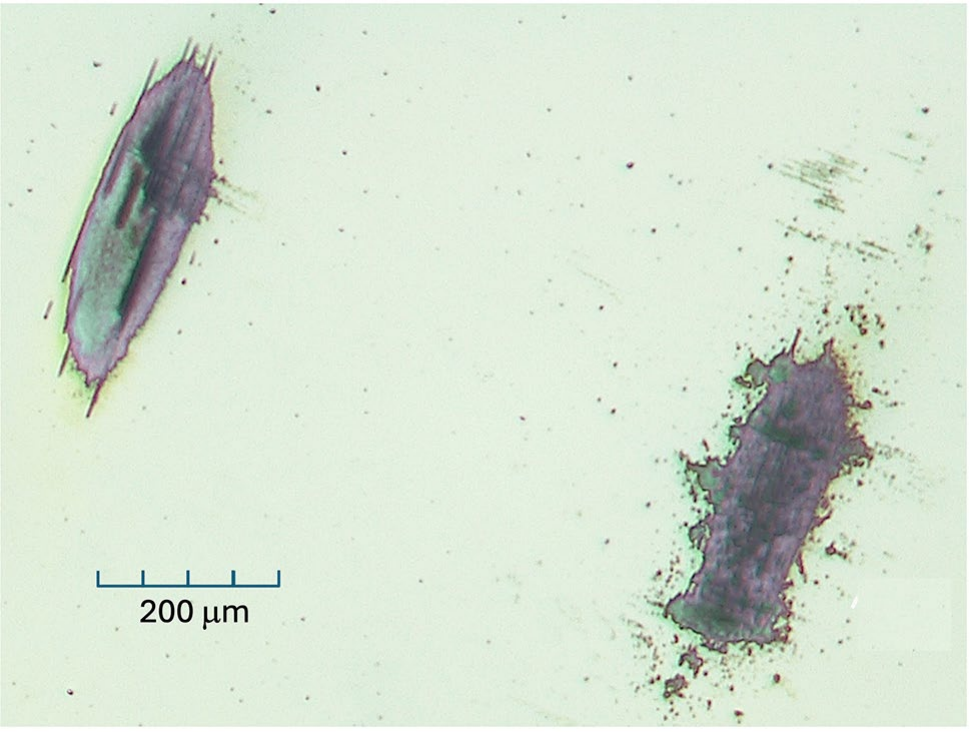
Optical micrographs of laser burn marks. The rough-shaped burn was obtained through the Mylar window of the sample chamber, giving rise to optical speckles.

However, at this fluence, burns were observed on the sample. Even with as low as 20% fluence (56 mJ/cm^2^, with ND filter), damage was still observed on the sample. The intensity dropped progressively during scans with the laser on. We translated the sample in steps of 1 mm between scans to avoid damaging the same spot on the thin film; however, a visible burn mark was seen after each scan. Later photographs show a clean-edged elliptical pit in the surface, shown in Fig. 5. Nevertheless, for each fresh position on the sample, a ~5% drop in diffracted intensity was seen over the course of each scan. This was mitigated by alternating “laser-on” and “laser-off” shots and subtracting the results to see the differences, which were less affected by the damage.

In an attempt to minimize the sample damage, we set up a helium environment, installing the chamber developed by Sogang University. We were concerned that the Mylar window in the chamber was changing the laser beam shape by introducing optical speckles (Fig. 5). However, it was not significantly reducing the fluence, with 50% laser fluence (250 mJ/cm^2^), because we still saw burn marks that were about the same size. Sample D was found to be stable, and we checked that the peak returned to the chosen spot on the detector.

**Fig. 6:**
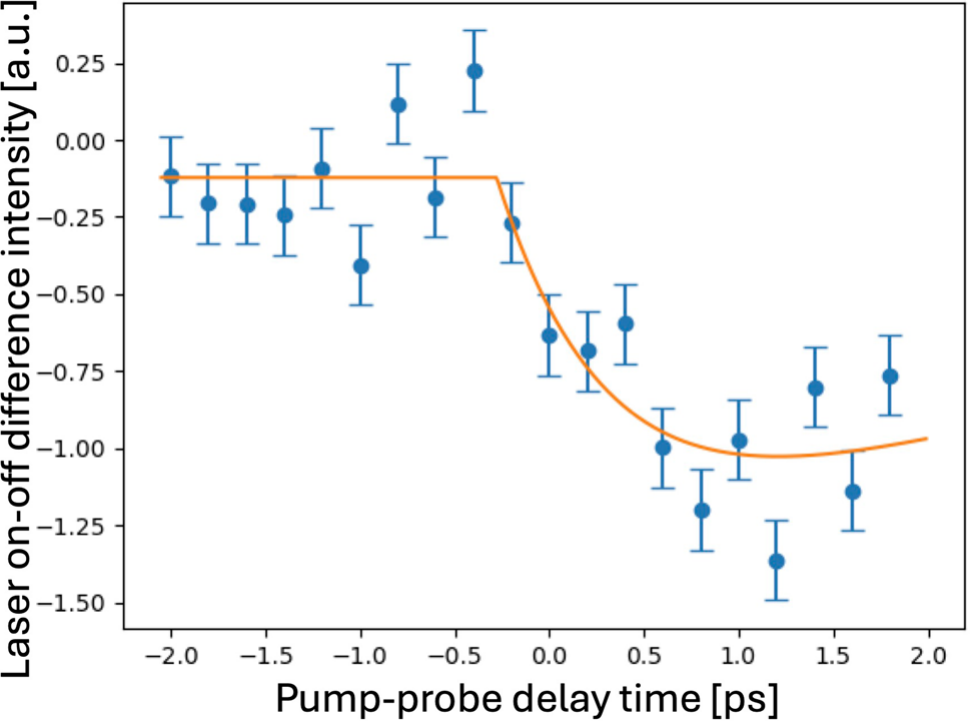
Fine time-step pump-probe (PP) scan of the 222 “forbidden” peak intensity versus time delay with 0.2 ps steps. These measurements were made on sample D with thickness 52 nm at a laser fluence of 20% (56 mJ/cm^2^, with ND filter). Systematic error bars are estimated to represent the overall reproducibility of the installation of the XFEL facility. The trend is fit to the sum of two [1−exp(−t/t_n_)] relaxation functions with opposite weights (to restore zero after long times). The time offset, −0.28 ps, corrects for the calibration of the temporal overlap. The second decay time constant, t_2_, was fixed at 5 ps because there are insufficient measurements to determine it, while the initial decay time constant, t_1_ = 0.626 ps, is well determined.

Once the damage behavior and the ways to avoid it were understood, we made some longer statistics runs (900 shots, or 30 s per point) very close to time zero to look for the melting of the valence electrons. Figure 6 shows a step scan over ~2 ps with steps of 0.2 ps. We can see a clear downward intensity jump at t = 0 ps, indicative of the expected electronic response, and the fit shown allows us to measure the relaxation time of t_1_ = 0.626 ps.

## Discussion and Conclusions

Despite the challenges of a limited-time experiment at an XFEL facility, we made successful measurements of the pump-probe (PP) time response of the forbidden 222 diffraction peak of a thin-film silicon sample to 400 nm laser excitation. The structure factor of that reflection originates mostly from the valence electrons, which can undergo melting on a faster time scale than the lattice. The relatively high-fluence (> 50 mJ/cm^2^) 400 nm laser excitation pumps a significant fraction of the electrons from the valence band into the conduction band.

The long-time acoustic response (Fig. 4) showed a 25 ps oscillation period, faster than the 40 ps expected for a round-trip sound wave crossing the 170 nm film. This could be an indication of laser-induced hardening of the bonds which would lead to steeper phonon dispersion curves [10], but it needs to be confirmed in a future experiment, most importantly as a function of laser fluence.

Close inspection of the Δt = 0 ps PP delay region in Fig. 6 shows a transient drop of 222 intensity with the “laser-on” signal subtracted from “laser-off” values on alternate shots to mitigate accumulating damage effects. Double exponential fits to the data in Fig. 6 gives an electronic response time of 626 fs. We note that the shape of the response in Fig. 6 is similar to that of the lattice temperature curve predicted by the TTM, where the rise time is given by the electron–phonon coupling rate as energy is transferred from the rapidly generated hot electron population to the crystal lattice. If the analogy is correct, the relaxation time still involves the lattice and does not correspond to the pure electronic process. According to Keating [14], there are contributions to the forbidden 222 intensity expected from anharmonic vibrations which break the symmetry of the core-electron cancellation in Fig. 1 and contribute to the 222 upon heating of the lattice. At room temperature, these contributions are important for Ge but an order of magnitude smaller than the electronic contributions in Si [14].

An alternative explanation for the t_1_ = 0.626 ps response time of the valence electrons seen by the Si(222) reflection is electron/hole diffusion. The laser is absorbed in the electromagnetic “skin depth” at the sample surface, which is around 100 nm for 400 nm excitation [8]. But at the relatively high fluence we found was needed to see an effect, the electrons become multiply excited and reach a very high temperature. These hot electrons then diffuse through the film, creating electron–hole pairs, of which the population of holes in the valence band is seen by the measurement. We can estimate the time for electrons/holes to diffuse across the thin film, a distance h = 52 nm from the surface, from the point-source (Green’s function) solution of the diffusion equation as t_D_ = h^2^/2D, where D is the diffusivity. Electrons or holes diffusing in a semiconductor have a diffusivity D = μ k_B_T/e, where μ is the mobility and e is the electronic charge. For electrons in silicon, μ_e_ = 1400 cm^2^/Vs, while for holes, μ_h_ = 450 cm^2^/Vs. At T = 300 K, this gives diffusion times t_D_ = 0.39 ps and 1.2 ps for electrons and holes, respectively. These times agree quite well with our measured number. Hot electrons would diffuse much faster but would cool down as they scatter along their path through the film. This mechanism might explain why the transient was only seen on the thinner sample D (Fig. 6), not the 170 nm film (Fig. 4), for which the diffusion times to fill the sample would be ten times longer, noting that the X-rays see an average signal from the entire sample thickness.

The femtosecond response time of semiconductors has been discussed extensively in the literature, long before XFELs were available to probe the atomic motions or valence electrons directly. Second-harmonic generation (SHG) pump-probe experiments date back to 1983 when the first femtosecond rhodamine lasers became available. Shank et al. saw an abrupt ~100 fs response in silicon at 200 mJ/cm^2^ fluence, followed by a slower second component [17]. SHG experiments on GaAs with 340 mJ/cm^2^ pumping [18] and Ge at 200 mJ/cm^2^ [19] followed, finding a similar 300 fs response time. The Ge results were interpreted as non-thermal (electronic) melting giving the fast component and melt-front propagation for the slower component [19]. The first x-ray diffraction experiments by Rousse et al. using a plasma x-ray source saw similar time scales in the 111 diffraction peak of InSb pumped at 120 mJ/cm^2^, which were also interpreted as non-thermal melting [9]. Pump-probe photoemission experiments on Si found a 100 fs carrier lifetime attributed to electron–phonon coupling [20]. Pump-probe electron diffraction experiments on thin polycrystalline Si films found 400 fs decay times at fluence of 65 mJ/cm^2^ [21] and recent x-ray pump x-ray probe experiments on Si were found to be consistent with an inertial acceleration model of the ionic displacements completed within 100 fs [22]. The implication is that the electron-lattice equilibration time in semiconductors (~100 fs) is an order of magnitude shorter for semiconductors than metals (~1 ps) in the TTM description. We must therefore consider that the t_1_ = 0.626 ps response time of the valence electrons seen in our experiment may include contributions from the non-thermal melting and shorter electron–phonon coupling times attributed to semiconductors.

## Data Availability

All data, code, and materials used for the completion of this manuscript are available from the corresponding author, IKR, upon request.

## References

[1] H. Ichikawa, S. Nozawa, T. Sato, A. Tomita, K. Ichiyanagi, M. Chollet, L. Guerin, N. Dean, A. Cavalleri, S. Adachi, T. Arima, H. Sawa, Y. Ogimoto, M. Nakamura, R. Tamaki, K. Miyano, and S. Koshihara, Transient photoinduced ‘hidden’ phase in a manganite. *Nat Matls* 10, 101 (2011). 10.1038/nmat2929 21240287

[2] O. Uteza, E. Gamaly, A. Rode, M. Samoc, and B. Luther-Davies, *PRB* 70, 054108 (2004).

[3] W. Hu, S. Kaiser, D. Nicoletti, C.R. Hunt, I. Gierz, M.C. Hoffmann, M. Le Tacon, T. Loew, B. Keimer, and A. Cavalleri, Optically enhanced coherent transport in YBa_2_Cu_3_O_6.5_ by ultrafast redistribution of interlayer coupling. *Nat. Matls.* 13(7), 705 (2014). 10.1038/nmat3963 24813422

[4] B. Abbey, R.A. Dilanian, C. Darmanin, R.A. Ryan, C.T. Putkunz, A.V. Martin, D. Wood, V. Streltsov, M.W.M. Jones, N. Gaffney, F. Hofmann, G. J. Williams, S. Boutet, M. Messerschmidt, M.M. Seibert, S. Williams, E. Curwood, E. Balaur, A. Peele, K.A. Nugent and H. Quiney, X-ray laser–induced electron dynamics observed by femtosecond diffraction from nanocrystals of Buckminsterfullerene. *Sci. Adv.* 2, e1601186 (2016). 27626076 10.1126/sciadv.1601186 PMC5017826

[5] S.I. Anisimov, B.L. Kapeliovich, T.L. Perelman, and L.D. Landau, Electron emission from metal surfaces exposed to ultrashort laser pulses. *Soviet J. Exp. Theor. Phys.* 39, 375–377 (1974).

[6] Y. Giret, N. Naruse, S.L. Daraszewicz, Y. Murooka, J. Yang, D.M. Duffy, A.L. Shluger, and K. Tanimura, Determination of transient atomic structure of laser-excited materials from time- resolved diffraction data. *Appl. Phys. Lett.* 103, 253107 (2013).

[7] J.N. Clark, L. Beitra, G. Xiong, A. Higginbotham, D.M. Fritz, H.T. Lemke, D. Zhu, M. Chollet, G.J. Williams, M. Messerschmidt, B. Abbey, R.J. Harder, A.M. Korsunsky, J.S. Wark, and I.K. Robinson, Ultrafast three dimensional imaging of lattice dynamics in gold nanocrystals. *Science* 341, 56 (2013). 23704372 10.1126/science.1236034

[8] http://www.pvlighthouse.com.au/Resources/Photovoltaic%20materials/Refractive%20index /Refractive%20index.aspx

[9] A. Rousse, C. Rischel, S. Fourmaux, I. Uschmann, S. Sebban, G. Grillon, Ph. Balcou, E. Förster, J.P. Geindre, P. Audebert, J.C. Gauthier, and D. Hulin, Non-thermal melting in semiconductors measured at femtosecond resolution. *Nature* 410, 65 (2001). 11242040 10.1038/35065045

[10] V. Recoules, J. Clérouin, G. Zérah, P.M. Anglade, and S. Mazevet, Effect of intense laser irradiation on the lattice stability of semiconductors and metals. *Phys. Rev. Lett.* 96, 055503 (2006). 16486947 10.1103/PhysRevLett.96.055503

[11] R. Ernstorfer, M. Harb, C.T. Hebeisen, G. Sciaini, T. Dartigalongue, and R.J.D. Miller, The formation of warm dense matter: experimental evidence for electronic bond hardening in gold. *Science* 323, 1033 (2009). 19164708 10.1126/science.1162697

[12] A. Descamps, B.K. Ofori-Okai, O. Bistoni, Z. Chen, E. Cunningham, L.B. Fletcher, N.J. Hartley, J.B. Hastings, D. Khaghani, M. Mo, B. Nagler, V. Recoules, R. Redmer, M. Schörner, D.G. Senesky, P. Sun, H. Tsai, T.G. White, S.H. Glenzer, and E.E. McBride, Evidence for phonon hardening in laser-excited gold using x-ray diffraction at a hard x-ray free electron laser. *Sci. Adv.* 10, 5272 (2024). 10.1126/sciadv.adh5272 PMC10857355 38335288

[13] Y. Ishida, T. Togashi, K. Yamamoto, M. Tanaka, T. Taniuchi, T. Kiss, M. Nakajima, T. Suemoto, and S. Shin, Non-thermal hot electrons ultrafastly generating hot optical phonons in graphite. *Nat. Sci. Rep.* 1, 64 (2011). 10.1038/srep00064 PMC3216551 22355583

[14] J.B. Roberto, B.W. Batterman, and D.T. Keating, Diffraction studies of the (222) reflection in Ge and Si: anharmonicity and the bonding electron. *Phys. Rev. B* 9, 2590 (1974).

[15] J. Ciston, A. Subramanian, I.K. Robinson, and L.D. Marks, Diffraction refinement of localized antibonding at the Si(111) 7x7 surface. *Phys. Rev. B* 79, 193302 (2009).

[16] R. Shayduk, J. Hallmann, A. Rodriguez-Fernandez, M. Scholz, W. Lu, U. Bosenberg, J. Moeller, A. Zozulya, M. Jiang, U. Wegner, R. Secareanu, G. Palmer, M. Emons, M. Lederer, S. Volkov, I. Lindfors-Vrejoiu, D. Schick, M. Bargheer, and A. Madsen, Femtosecond x-ray diffraction study of multi-THz coherent phonons in SrTiO_3_. *Appl. Phys. Lett.* 120, 202203 (2022).

[17] C.V. Shank, R. Yen, and C. Hirlimann, Femtosecond-time-resolved surface structural dynamics of optically excited silicon. *Phys. Rev. Lett.* 51, 900 (1983).

[18] K. Sokolowski-Tinten, J. Bialkowski, and D. von der Linde, Ultrafast laser-induced order-disorder transitions in semiconductors. *Phys. Rev. B* 51, 14186 (1995). 10.1103/physrevb.51.14186 9978346

[19] K. Sokolowski-Tinten and D. von der Linde, Ultrafast phase transitions and lattice dynamics probed using laser-produced x-ray pulses. *J. Phys. Condens. Matter* 16, R1517 (2004).

[20] H. Tanimura, J. Kanasaki, K. Tanimura, J. Sjakste, and N. Vast, Ultrafast relaxation dynamics of highly excited hot electrons in silicon. *Phys. Rev. B* 100, 035201 (2019).

[21] M. Harb, R. Ernstorfer, C.T. Hebeisen, G. Sciaini, W. Peng, T. Dartigalongue, M.A. Eriksson, M.G. Lagally, S.G. Kruglik, and R.J.D. Miller, Electronically driven structure changes of Si captured by femtosecond electron diffraction. *Phys. Rev. Lett.* 100, 155504 (2008). 18518123 10.1103/PhysRevLett.100.155504

[22] I. Inoue, V. Tkachenko, Y. Kubota, F. Dorchies, T. Hara, H. Höppner, Y. Inubushi, K.J. Kapcia, H.-J. Lee, V. Lipp, P. Martinez, E. Nishibori, T. Osaka, S. Toleikis, J. Yamada, M. Yabashi, B. Ziaja, and P.A. Heimann, Interplay of thermal and nonthermal effects in x-ray-induced ultrafast melting. *Phys. Rev. B* 110, L100102 (2024).

